# Development of Automatic Method for Glucose Detection Based on Platinum Octaethylporphyrin Sol–Gel Film with Long-Term Stability

**DOI:** 10.3390/s25010186

**Published:** 2024-12-31

**Authors:** Yujie Niu, Yongda Wang, Lu Li, Xiyu Zhang, Ting Liu

**Affiliations:** 1 Department of Physics, Northeast Forestry University, Harbin 150040, China; niuyujie2000@163.com; 2 School of Instrumentation Science and Engineering, Harbin Institute of Technology, Harbin 150001, China; ydw9180@163.com; 3 School of Physics, Harbin Institute of Technology, Harbin 150001, China; 22b311005@stu.hit.edu.cn; 4 College of Physical Science and Technology, Heilongjiang University, Harbin 150080, China; xyzcmp@163.com

**Keywords:** Pt/TE-MTS film, glucose concentration (GC), oxygen molecule, phosphorescence, aqueous solution

## Abstract

In this study, an approach has been proposed in response to the urgent need for a sensitive and stable method for glucose detection at low concentrations. Platinum octaethylporphyrin (PtOEP) was chosen as the probe and embedded into the matrix material to yield a glucose-sensing film, i.e., Pt/TE-MTS, through a sol–gel process. The optical parameter (OP) was defined as the ratio of phosphorescence in the absence and presence of glucose, and the relationship between OP and glucose concentration (GC) was established in a theoretical way based on the Stern–Volmer equation and further obtained by photoluminescence measurement. OP exhibited a linear relationship with GC in a range of 0–720 μM. The time required by the photoluminescence of the film to reach equilibrium was measured to ensure the completion of the reaction, and it was found that the equilibrium time decreased as the GC increased. The photobleaching behavior and stabilization of the film were monitored, and the result showed that the film exhibited excellent resistance to photobleaching and was quite stable in an aqueous solution. Additionally, a LabVIEW-based GC-detection system was developed to achieve the practical application of the sensing film. In summary, the Pt/TE-MTS film exhibited high sensitivity in detecting the GC with excellent reproducibility, which is of high value in applications.

## 1. Introduction

Glucose, a polyhydroxy aldehyde, is widely distributed in nature and plays a crucial role in diverse fields such as energy storage and metabolism in biosomes, the food industry, pharmaceuticals, biotechnology, and environmental sciences [1,2,3]. The concentration of glucose (GC) is usually regarded as a vital identifier, which helps us keep track of the correlated process. Therefore, monitoring the GC is significant for studying any process in which glucose is involved [4]. A sensitive and accurate method for detecting the GC has been under development for a long time, especially one for detecting a low GC [5]. A wide variety of methods were used for GC detection, such as electrochemical methods, mass spectrometry, enzymatic assays, chemical analyses, optical methods, and others [6,7,8,9]. Among these methods, the optical technique has garnered significant attention due to its advantages of high sensitivity, ease of operation, broad applicability, and exceptional accuracy [10,11].

Glucose oxidase (GOD) was used as a catalyst in the optical method for glucose concentration measurement. When oxygen molecules and glucose react in the presence of the catalyst GOD, the following redox reaction occurs: C_6_H_12_O_6_+O_2_→C_6_H_12_O_7_+H_2_O_2_ [12]. In the above reaction, the consumption of oxygen molecules is correlated with the concentration of glucose. Therefore, the detection of GC can be achieved by measuring the consumption of oxygen molecules indirectly [13]. Based on the above reaction, embedding an oxygen-sensitive probe that shows luminescence dependent on the concentration of oxygen molecules into a suitable supporting material can attain the effective monitoring of GC, but the chemical stability of the glucose-sensing material as well as the valid detection range of GC still need to be further explored and improved [14]. Metalloporphyrin represents a series of compounds formed by porphyrins and their derivatives with metal ions [15]. Among these compounds, platinum octaethylporphyrin (PtOEP) is confirmed to be extremely sensitive to oxygen molecules with strong phosphorescence emission and a stable chemical structure [16,17]. Due to its advantageous properties, PtOEP has been extensively used in the detection of oxygen concentration [18]. Encapsulating the probe molecules into an appropriate supporting material provided a better surrounding for storing the probe by extending its service life, reducing the photobleaching effect of the probe, and keeping it stable up to its next use [19,20,21,22]. Commonly used supporting materials include ethyl cellulose (EC), polystyrene (PS), polymethyl methacrylate (PMMA), sol–gel materials, and others [23,24,25,26]. Among these materials, sol–gel materials have relatively big air voids, which provide sufficient space for oxygen molecules to pass through [27]. Thus, oxygen molecules can establish contact with probe molecules rapidly, which makes achieving the detection of low GC easier. In addition, by adjusting the mechanical properties of the sol–gel materials, their hydrophobicity can be increased [28], resulting in a supporting material that is more stable in an aqueous solution. Finally, it is possible to achieve a method for low-GC detection based on a PtOEP-embedded sol–gel sensing film.

In this study, PtOEP and sol–gel were chosen as the oxygen-sensitive molecule and supporting matrix, respectively, to yield the Pt/TE-MTS film. The sensing film was prepared based on the spin-coating method. The UV–visible absorption spectra and photoluminescence spectra were measured to study the optical properties of this film. The hydrophobicity of the sensing film was analyzed by measuring the hydrophobic angle. The morphology of the film was characterized by scanning electron microscopy (SEM). The relationship between OP and GC was established in a theoretical way based on the Stern–Volmer equation. The photoluminescence spectra at different GCs were measured to explore the valid detection range of GC based on the Pt/TE-MTS film. The time required by the phosphorescence intensity of the film to reach its maximum value was determined to obtain the response time of the sensing film. Additionally, the photostability of the film was tested by the prolonged irradiation of pure water using a 405 nm commercial laser. The influence of pH and ions of the glucose solution on the GC measurements was assessed. Furthermore, the films were immersed in a solution of different pH values, i.e., in a solution containing ions for 10 days and in pure water for one month, to measure the stability of the films. Finally, a GC-detection system utilizing the optical method was developed using the LabVIEW software (version: 2018). The application of this system was assessed using real samples. The developed sensing film enabled the detection of low GC, which has potential applications in the fields of biomedical monitoring and food science.

## 2. Materials and Methods

### 2.1. Reagents and Materials

Tetraethyl orthosilicate (TEOS) and triethoxymethylsilane (MTES) were provided by Shanghai Aladdin Biochemical Technology Co., Ltd. (Shanghai, China). Glucose oxidase (GOD, 100 U/mg) and platinum octaethylporphyrin (PtOEP) were obtained from Shanghai Macklin Biochemical Technology Co., Ltd. Toluene (Shanghai, China), ethanol (EtOH) and deionized water were supplied by Xilong Reagent Co., Ltd. (Shantou, China). Ammonium hydroxide and hydrochloric acid (HCl) were provided by Shanghai Aladdin Biochemical Technology Co., Ltd. Glucose injection (100 mL:5 g) was obtained from Harbin Medisan Pharmaceutical Co., Ltd. (Harbin, China). All reagents were utilized as received without further purification.

### 2.2. Sample Preparation

TEOS (1.4 mL) and MTES (1 mL) were mixed on a magnetic stirrer at 300 rmp to prepare the precursor solution. Subsequently, deionized water (0.5 mL), EtOH (3.5 mL), and HCl (10 μL) were added to the precursor solution to catalyze the ORMOSILs for hydrolysis [29]. The mixture was stirred for 40 min. NH_4_OH was then added to the mixture to adjust the PH of the solution. At this stage, the sol solution was successfully obtained. PtOEP was dissolved in toluene to prepare a luminophore-solution, which was subsequently added to the sol solution in a 1:4 ratio to yield a luminophore-sol solution via the sol–gel process technique. The luminophore-sol solution was spin-coated onto a glass slide that had been cleaned with NaOH and EtOH, and dried at room temperature for 2 h. Finally, the Pt/TE-MTS film was obtained after further drying in an oven at 30 °C for 48 h.

### 2.3. Instruments and Characterization

The surface and cross-sectional morphology of the film was characterized using scanning electron microscopy (SEM, JSM–7500F, JEOL Ltd., Akishima, Japan). The hydrophobicity of the sensing membrane was evaluated using a contact angle measuring instrument (SDC–350, SINDIN, Dongguan, China). The UV–visible absorption spectra were measured using a deuterium lamp as the light source and recorded by a miniature fiber optic spectrometer (QE65000, Ocean Optics, Orlando, FL, USA). The photoluminescence spectra were recorded using a miniature fiber optic spectrometer (USB2000, Ocean Optics) equipped with a 405 nm laser as the excitation source. The UV–vis absorption spectra of PtOEP and photoluminescence spectra of PtOEP/sol–gel were measured to study the optical properties relevant to the sensing film.

To achieve the relationship between OP and GC based on the sensing film, the film was secured in a sealed device equipped with two ports: one for controlling the inflow of glucose solution at various concentrations and the other for introducing glucose oxidase (GOD). The measurements were conducted under fully sealed conditions. Initially, 1.6 mL glucose solution at various concentrations was added to the sealed device, followed by 400 μL of GOD solution (2.5 mg/mL). The time required by the phosphorescence intensity of the film to reach its maximum was determined by monitoring the phosphorescence intensity every 40 s using a 405 nm laser as the light source, until the phosphorescence intensity was stabilized, and the time was obtained. This measurement procedure was repeated at intervals of 120 μM within a GC range of 0–1080 μM.

The photobleaching behavior was analyzed by continuously irradiating the film for 4000 s using a 405 nm laser with a power of 1.5 mW/cm^2^. To further examine the stability of the sensing film, it was immersed in deionized water, an ionic liquid (NaCl solution), and solutions with pH values of 6 and 7.5 for reliability testing. Photographs were taken of the films immersed in deionized water for one month, in solutions with pH values of 6 and 7.5, and in the ionic liquid for 10 days. For comparison, Pt/EC and Pt/PMMA films immersed in deionized water were also tested. The phosphorescence of the films, which had been immersed in deionized water for one month and in other solutions for 10 days, was measured over a GC range of 0–1080 μM at intervals of 120 μM to determine the relationship between OP and GC.

The effects of pH and ions on GC measurements were evaluated. The phosphorescence of the films was measured at various GC levels under the pH values of 6, 6.5, 7, 7.5, and 8 to establish the correlation between OP and GC. Solutions containing Na^+^, K^+^, Cl^−^, HCO_3_^−^, and Ca^2+^ ions were prepared by adding these ions to the glucose solution. OP values were measured in solutions with different ions at GC levels of 150 μM, 300 μM, and 500 μM. Additionally, the blank glucose solution without any ions was measured as the controls.

To evaluate the accuracy of the glucose detection system, glucose injection (100 mL:5 g) was diluted to final concentrations of 100, 250, 400, 550, and 700 μM for testing. The measured GC values were obtained using the system, and the relative errors for each GC level were calculated to assess the accuracy of the glucose detection system.

## 3. Results and Discussion

### 3.1. Morphology and Contact Angle of the Pt/TE-MTS Film

**Figure 1:**
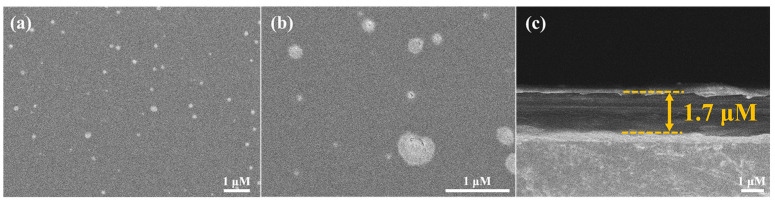
SEM images of the Pt/TE-MTS film: (**a**,**b**) surface morphology; (**c**) cross-sectional morphology.

A scanning electron microscope was employed to observe the microstructure of the Pt/TE-MTS film. The surface and cross-sectional morphology of the Pt/TE-MTS film were characterized, as shown in Figure 1. Figure 1a exhibits the surface morphology of the Pt/TE-MTS film, revealing a flat and smooth surface with an evident porous structure. To further examine the characteristics of the pores, Figure 1b provides a localized magnification of Figure 1a, showing that the average diameter of the pores is approximately 0.18 µm, which facilitates the diffusion of oxygen molecules (approximately 0.3 nm). This indicates that the dissolved oxygen can rapidly and thoroughly interact with PtOEP embedded into the film. Further observation of the cross-sectional morphology in Figure 1c reveals a film thickness of 1.7 µm, confirming its micromere-scale dimensions.

**Figure 2:**
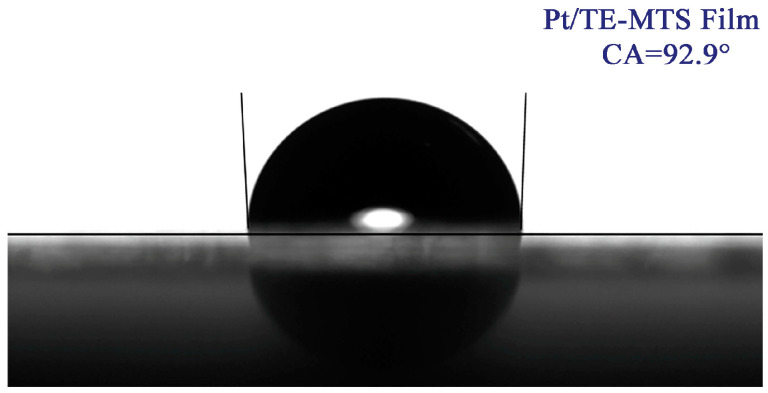
The contact angle of the Pt/TE-MTS film.

The contact angle of the Pt/TE-MTS film was characterized and is shown in Figure 2. The contact angle of the Pt/TE-MTS film was measured to be 92.9°, which exceeds the critical contact angle of 90° [30,31], indicating that the Pt/TE-MTS film exhibits hydrophobic properties. Given that PtOEP demonstrates poor stability in aqueous solutions [32], the hydrophobic nature of the film provides adequate protection and an appropriate medium for PtOEP to detect glucose in aqueous environments. The results from the SEM analysis and contact angle measurement indicate that the Pt/TE-MTS film not only provides superior protection for PtOEP but also facilitates rapid and efficient glucose detection.

### 3.2. Optical Properties of the Pt/TE-MTS Film

**Figure 3:**
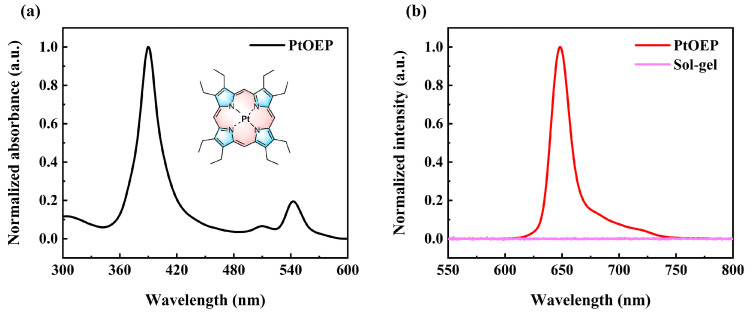
(**a**) Normalized UV–absorption spectrum of PtOEP/methanol. Inset: chemical structure of PtOEP; (**b**) normalized photoluminescence spectrum of PtOEP/methanol and supporting matrix (TEOS–MTES).

The optical properties of the film are crucial for its application, with PtOEP serving as the oxygen indicator and the sol–gel material acting as the supporting matrix. In this study, the typical UV–visible absorption spectrum and photoluminescent spectrum were measured to investigate the optical characteristics of the probe and the supporting matrix, as shown in Figure 3. The normalized UV–visible absorption spectrum of PtOEP is shown in Figure 3a, with the chemical structure of PtOEP provided in the inset of Figure 1a. Two weak absorptions at 510 nm and 543 nm, corresponding to the Q–bands [33], and a strong absorption at 389 nm, which belongs to the Soret band [34], were observed in the spectrum. Consequently, a 405 nm laser was selected as the excitation source to obtain the photoluminescence spectrum. The normalized photoluminescence spectra of PtOEP (red line) and TEOS–MTES supporting matrix (pink line) are shown in Figure 3b. A strong photoluminescence peak centered at 648 nm was observed in the photoluminescent spectrum of PtOEP, confirming it as a phosphorescence peak. Additionally, the TEOS–MTES supporting matrix exhibits no luminescence under the excitation of a 405 nm laser, as illustrated in Figure 3b.

### 3.3. The Theoretical Relationship Between OP and GC

**Figure 4:**
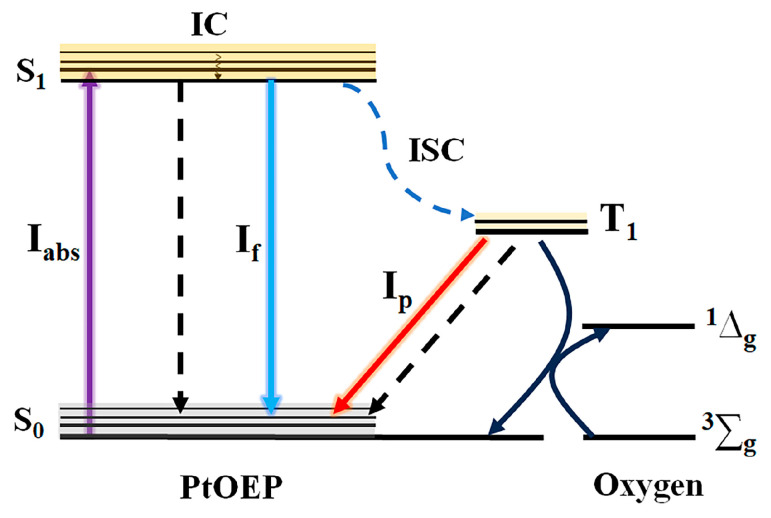
Energy level schematic diagram of PtOEP.

To investigate the correlation between GC and the phosphorescence of PtOEP, the potential energy transfer processes were analyzed, as illustrated by the energy level scheme in Figure 4. When a PtOEP molecule is excited by a 405 nm laser, it transfers from the ground state (S_0_) to a higher singlet excited state, subsequently relaxing to the bottom of the first singlet excited state (S_1_). At the T_1_ state, PtOEP has three possible decay pathways: (i) returning to S_0_ via a non–radiative pathway; (ii) fluorescence emission (I_f_); and (iii) it may transfer to the first excited triplet state (T_1_) by means of the intersystem crossing process [35]. PtOEP at T_1_ state can return to S_0_ through a phosphorescence (*I*_p_) and a non-phosphorescence way; additionally, it can also return to S_0_ via interacting with O_2_. Therefore, the phosphorescence intensity of PtOEP can be effectively quenched by oxygen molecules in the environment.

The quantitative relationship between phosphorescence and oxygen concentration was described using the Stern–Volmer equation [36], which is expressed as follows:

(1) $$\frac{I_{P0}}{I_{P}}=1+K_{\mathrm{SV}}[O_{2}]$$

where *I*_P0_ is the phosphorescence intensity in the absence of oxygen, *I*_P_ is the phosphorescence intensity in the presence of oxygen, and [*O*_2_] is the oxygen concentration of the substance to be measured. *K*_SV_ is the constant for the phosphorescence to be quenched by oxygen, which represents the sensitivity of an oxygen sensor. In a sealed container, when glucose and GOD are both present [37,38], a redox reaction occurs as follows:

(2) $$C_{6}H_{12}O_{6}+O_{2}\overset{\mathrm{GOD}}{\to }C_{6}H_{12}O_{7}+H_{2}O_{2}$$

Due to the consumption of oxygen, the phosphorescence of the Pt/TE-MTS film changes, which can be described by Equation (1). Once the reaction reaches equilibrium, the phosphorescence remains constant. The ratio of phosphorescence in the absence and presence of glucose (OP) can be derived as follows:

(3) $$\mathrm{OP}=\frac{I_{G0}}{I_{\mathrm{Gq}}}=1-S\;[\Delta O_{2}]$$

where *I*_G0_ and *I*_Gq_ are the phosphorescence intensities in the absence and presence of oxygen, respectively. [Δ*O*_2_] represents the amount of oxygen consumed in the sealed container. *S* is the parameter correlated with the consumption of [Δ*O*_2_]. Based on the redox reaction described in Equation (2), the consumption of oxygen is equivalent to that of glucose. Therefore, the oxygen consumption can be replaced by the glucose consumption. Equation (3) can be expressed as follows:

(4) OP=1−S [ΔG]

Here, *S* is related to the glucose consumption rate, representing the sensitivity for glucose measuring.

### 3.4. The Relationship Between OP and GC Based on Pt/TE-MTS Film

**Figure 5:**
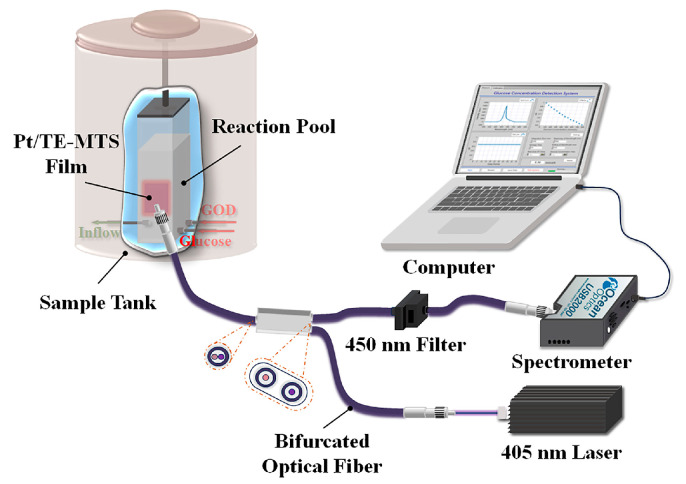
Glucose concentration detection system.

To further obtain the correlation between phosphorescence and glucose based on the Pt/TE-MTS film, a series of devices were planned, as shown in Figure 5.

**Figure 6:**
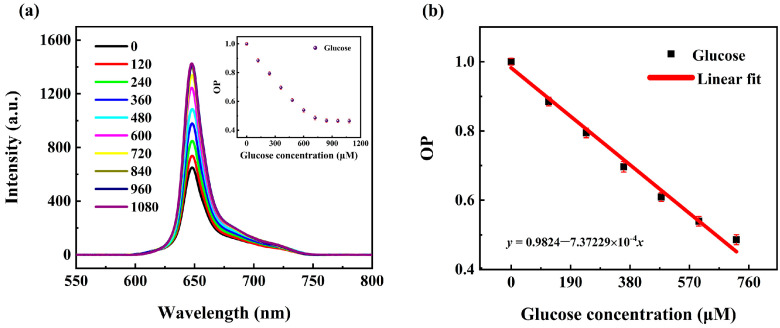
(**a**) Luminescence spectra of the sensing film at different glucose concentrations. Insert: the relationship between OP and glucose concentrations. (**b**) OP at different glucose concentrations in a range of 0–720 μM.

Based on the theoretical relationship described in Equation (4), the phosphorescence intensity of the Pt/TE-MTS film was measured at various GCs, as illustrated in Figure 6a. It was observed that the profile of the phosphorescence remained consistent across different GCs. The phosphorescence intensity increased significantly with increasing GC in a range of 0–720 μM. However, when GC exceeded 720 μM, the phosphorescence intensity remained nearly constant. This phenomenon is related to the oxygen content in the sealed container. According to the redox reaction process described in Equation (2), glucose and oxygen react sympathetically within the container. When the GC is relatively low, the content of oxygen in the sealed container is sufficient for the complete redox reaction. As GC increases, the consumption of oxygen also increases, leading to a subsequent increase in phosphorescence intensity. However, when the GC exceeds 720 μM, the content of oxygen becomes minimal and insufficient to support further reaction, resulting in the exhausting of oxygen, and the phosphorescence intensity remains constant. The correlation between OP and GC is shown in Figure 6b. It was observed that OP exhibited a linear relationship with GC in a range of 0–720 μM, and then remained constant as GC increased. By linear-fitted the experimental data, the quantitative linear relationship between OP and GC was obtained as follows:

(5) $$\mathrm{OP}=0.98-7.37\times 10^{-4}[G]$$

The relationship derived from the experimental data based on Pt/TE-MTS film is consistent with the theoretical relationship established in Equation (4). Furthermore, this relationship can serve as a foundation for glucose detection using the Pt/TE-MTS film, allowing GC to be indirectly determined through phosphorescence measurement. This methodology has been generally confirmed to exhibit high sensitivity and accuracy. Meanwhile, the limit of detection, based on the linear-fitted results and noise analysis, was approximately 46 μM (S/N = 3), which is lower than that reported in the relevant studies [39,40,41,42].

### 3.5. Equilibrium Time for the Pt/TE-MTS Film

**Figure 7:**
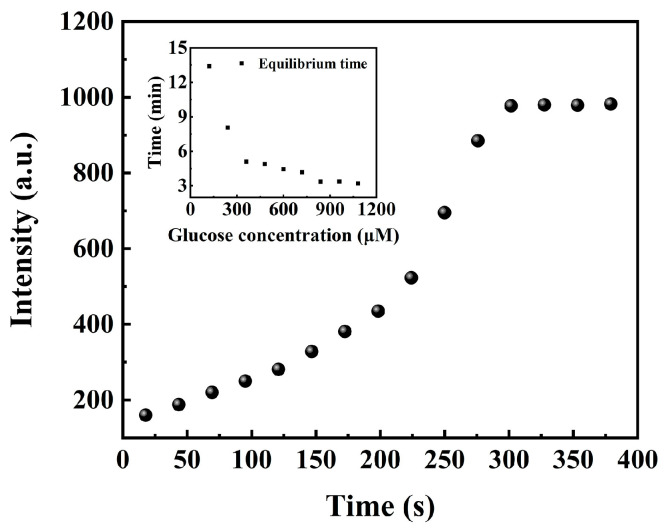
Phosphorescence intensity of the film in glucose concentration of 360 μM with time increased. Inset: time required for phosphorescence intensity to reach its maximum in glucose concentration of 0–1080 μM.

It takes time for the redox reaction which involves glucose, oxygen, and GOD to carry on. To accurately measure the phosphorescence intensity at various GCs, it is necessary to determine the time required for the redox reaction to reach completion. The phosphorescence of the Pt/TE-MTS film was monitored with a fixed amount of glucose (360 μM) stored in a sealed container, as shown in Figure 7. It was observed that the phosphorescence intensity increased significantly within the first 300 s, after which the phosphorescence intensity tended to be stabilized. Based on this observation, the reaction was confirmed to be complete at 300 s. To obtain the equilibrium times for various GCs involved in Figure 6, the phosphorescence of the Pt/TE-MTS film was measured over the increasing reaction times for GCs ranging from 0 to 1080 μM, as shown in the inset of Figure 6. It was found that the equilibrium time decreased as the GC increased. This behavior could be attributed to the enhanced interaction between glucose and GOD, leading to faster oxygen consumption and quicker stabilization of phosphorescence. All phosphorescence measurements of the Pt/TE-MTS film in Figure 6 were obtained after the reaction had fully completed in the sealed container to ensure accurate glucose detection.

### 3.6. Photobleaching Resistance of the Pt/TE-MTS Film

Photobleaching is generally caused by the irreversible photochemical reaction of the luminophore when it is exposed to light and is widely observed in the application of luminescent materials. Due to the photobleaching, the luminescence intensity of luminophores progressively decreases or may even vanish with prolonged light exposure. This can lead to a reduction in the signal-to-noise ratio, affect the precision of the results, and reduce the longevity of the material. Therefore, evaluating the photobleaching behavior of the Pt/TE-MTS film is essential for its practical application.

**Figure 8:**
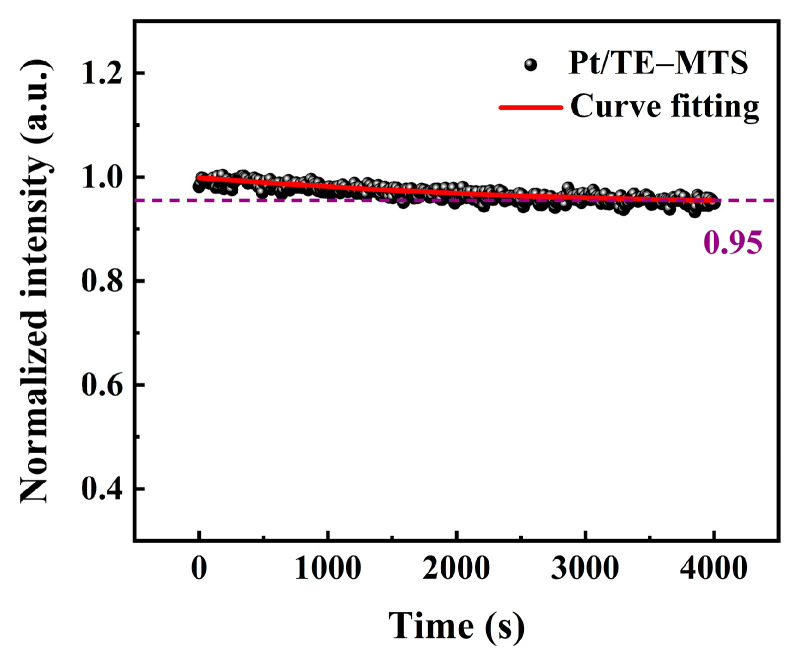
Luminescence intensity at 648 nm of the sensing film after 4000 s of continuous irradiation.

To test the photobleaching behavior of the sensing film, the film was continuously irradiated using a 405 nm commercial semiconductor laser with a power density of 1.5 mW/cm^2^. As shown in Figure 8, the phosphorescence intensity of the sensing film remained above 95% after 4000 s of continuous laser irradiation, indicating that the film can maintain stable phosphorescence emission over an extended period. Obviously, the sensing film exhibits excellent light resistance, ensuring reliable detection in complex environments with a high signal-to-noise ratio.

### 3.7. Effect of Interfering Ions and pH on Pt/TE-MTS Film

Typically, the pH value of the testing solution is variable, and the presence of certain ions in the testing environment may interfere with the accurate quantification of glucose. Therefore, it is imperative to evaluate the specificity of the Pt/TE-MTS film in complex environments.

**Figure 9:**
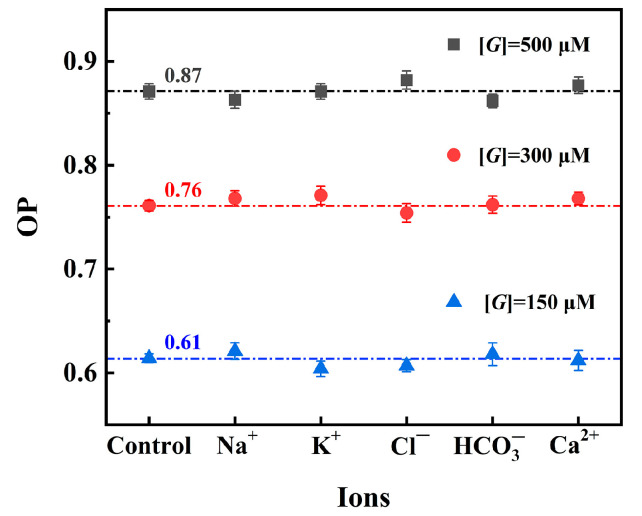
OP values in solutions with GC of 150, 300, and 500 μM containing various interfering ions, blank glucose solution at the same GC was defined as the control group.

To evaluate the effect of the interfering ions on glucose detection using Pt/TE-MTS film, several ions (Na^+^, K^+^, Cl^−^, HCO_3_^−^, and Ca^2+^) were selected as potential interferents for testing. Figure 9 exhibits the OP values in solutions with GC of 150, 300, and 500 μM containing various interfering ions, the blank glucose solution at the same GC was used as the control group for comparison. The OP values in the glucose solutions containing interfering ions were found to be consistent with those in the blank glucose solution at each GC. The results indicate that the detection of glucose based on the Pt/TE-MTS film is not influenced by potential interfering ions.

**Figure 10:**
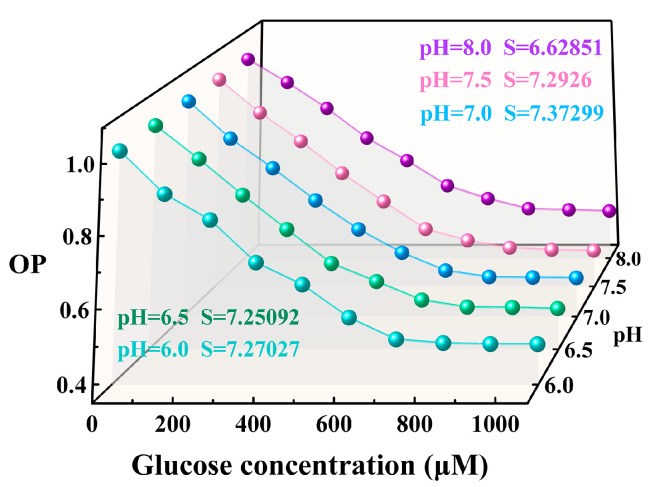
OP values dependence on glucose concentration in the pH range of 6–8.

Furthermore, we studied the effect of pH on glucose determination using the Pt/TE-MTS film. Figure 10 exhibits the dependence of OP on GC in the pH range of 6–8. The results show that OP exhibits a consistent change trend with the increasing GC across various pH values of the solution. In a GC range of 0–720 μM, OP shows a linear relationship with the increasing GC and then kept invariable in different pH values of the solution. By linear-fitted the experimental data in the GC range of 0–720 μM, the sensitivity *S* for glucose measurement based on the Pt/TE-MTS film, as descripted in Equation (4), was obtained. The results indicate that *S* remains consistent in the pH range of 6.0–7.5; however, when the pH value of the solution reaches 8, *S* decreases significantly. This decrease is attributed to the reduced activity of glucose oxidase at higher pH values [43]. These findings suggest that the Pt/TE-MTS film exhibits excellent performance in glucose detection when the pH level is below 8.

### 3.8. Stability of the Pt/TE-MTS Film

**Figure 11:**
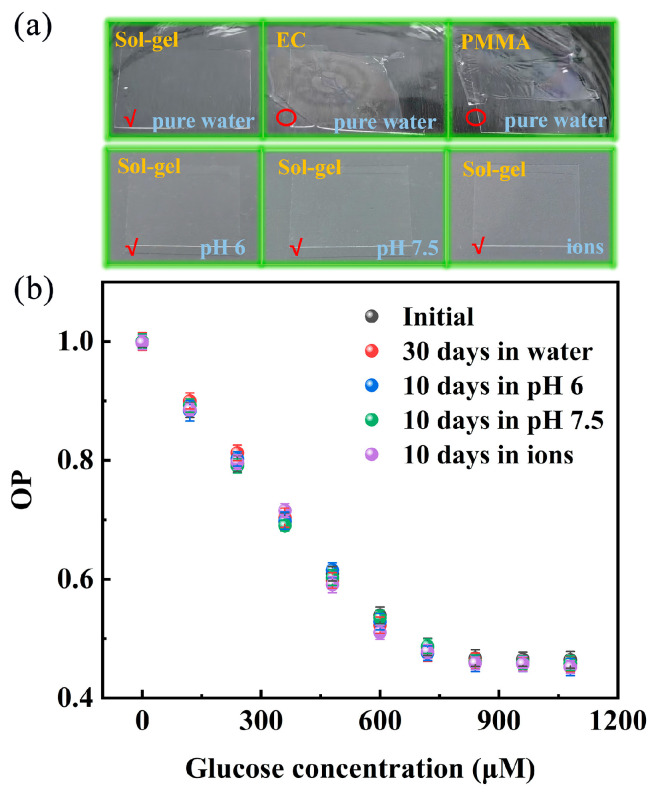
(**a**) Images of Pt/TE-MTS film immersed in various solutions for a period of time, with Pt/EC film and Pt/PMMA film immersed in pure water for comparison; (**b**) the dependence of OP on glucose concentration for the films immersed in various solutions for a period of time compared with the unsoaked film.

The robustness and stability of the Pt/TE-MTS film in complex environments are critical for its practical application. Therefore, the stability of the Pt/TE-MTS film was evaluated in various solutions. The film was immersed in different solutions for specified periods: one month in pure water, 10 days in solutions with pH values of 6 and 7.5, and 10 days in solutions containing ions. Furthermore, two commonly used matrices for glucose detection (EC and PMMA) were combined with PtOEP separately for comparison, as shown in Figure 11a. It can be observed that the Pt/TE-MTS film remains firmly adhered to the glass sheet after being immersed in pure water for one month, while both the Pt/EC film and Pt/PMMA film were significantly detached from the glass. Additionally, the Pt/TE-MTS film remained tightly adhered to the glass sheet after immersion in solutions containing ions as well as in pH values of 6 and 7.5 for 10 days. Furthermore, the sensitivity of the Pt/TE-MTS film for GC detection was also verified. The relationship between OP and GC for the immersed films was monitored and compared with that of the unsoaked sample, as shown in Figure 11b. It was found that in the GC range of 0–720 μM, all immersed films exhibited a linear relationship between OP and GC, which then remained constant, consistent with the unsoaked sample. These results indicate that Pt/TE-MTS film demonstrates high stability and robustness in complex environments for continuous glucose detection.

### 3.9. Glucose Sensing System

**Figure 12:**
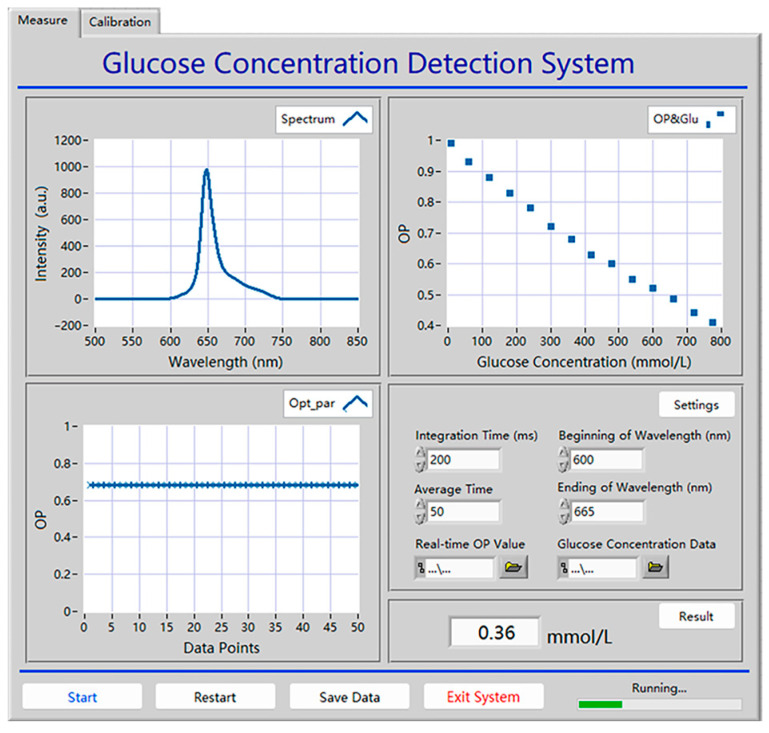
The front panel of the glucose sensing system.

**Figure 13:**
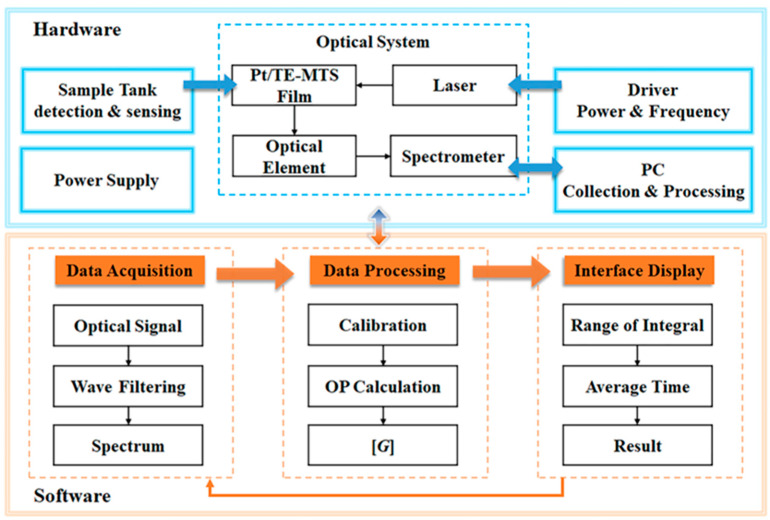
Principle block diagram of the glucose sensing system.

To extend the scope of application for glucose detection using the Pt/TE-MTS film, a system based on LabVIEW was developed to realize the GC measurement. The display panel of the detection system is shown in Figure 12. The system primarily consists of three modules: a spectra testing module, a data recording and analysis module, and a parameter setting module. The emission spectrum is recorded within a wavelength range of 500–850 nm, and the OP value is calculated through multiple data acquisitions; the linear relationship between the OP value and the corresponding GC is displayed. Moreover, the integration time, average time, and the wavelength range of integration for the system could be easily adjusted by the users. The principle block diagram of the glucose detection system is shown in Figure 13. Furthermore, the system incorporates data storage and analysis features to enhance usability. Based on this system, GC can be automatically detected in real-time.

### 3.10. Application of Glucose Sensing System Based on Pt/TE-MTS Film

**Figure 14:**
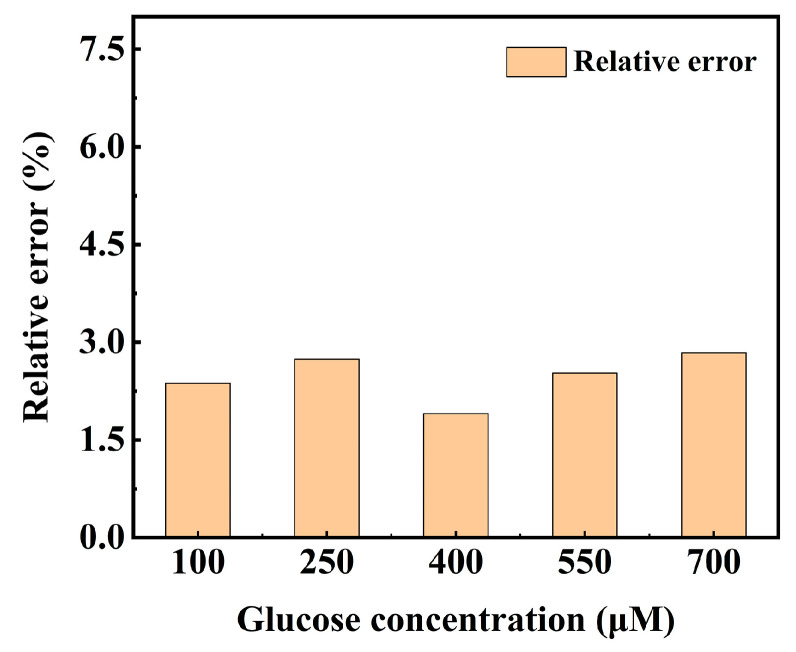
Relative errors for the samples with glucose concentrations of 100, 250, 400, 550, and 700 μM.

To validate the accuracy of the glucose detection system based on the Pt/TE-MTS film designed with LabVIEW, glucose injections with known concentrations were prepared for testing. The glucose solution was diluted to achieve final concentrations of 100, 250, 400, 550, and 700 μM, respectively. The measured GC values for these samples were obtained using the glucose sensing system. By comparing the measured GC values with the real GC values of the samples, the accuracy of the glucose detection system can be evaluated. The relative error was used to assess the accuracy of the system, and is formally defined as the following Equation.

(6) $$\delta =\left(\frac{\left|{\left[G\right]}_{M}-{\left[G\right]}_{R}\right|}{{\left[G\right]}_{R}}\right)\times 100\%$$

where *δ* is the relative error, [*G*]_M_ represents the measured GC values obtained by the system, and [*G*]_R_ represents the real GC values of the samples. The relative errors for the samples with GC of 100, 250, 400, 550, and 700 μM are shown in Figure 14. It can be observed that there is only a minor difference between the measured and the real values for all the samples, and the relative errors for the measured samples were below 3%. The OP values and measured GC values obtained by the system, along with the calculated relative errors are presented in Table 1. These results verify that the system has the capability to measure glucose concentration with minimal relative error.

## 4. Conclusions

In this work, the Pt/TE-MTS film was developed for glucose concentration (GC) detection using a phosphorescence quenching mechanism. TEOS-MTES was chosen as the matrix material and PtOEP was used as the oxygen-sensitive molecule. SEM results showed that the film was smooth and porous, facilitating the transport of oxygen molecules. The contact angle measurement of 92.9° indicated that the film exhibited hydrophobic properties. A theoretical relationship between OP and GC was established as OP = 1 − *S*Δ[*G*]. The experimental results demonstrated that OP exhibited a linear relationship with GC in the range of 0–720 μM. The limit of detection was determined to be 46 μM. In addition, the equilibrium time for the complete reaction decreased as the GC increased. After continuous irradiation by a 405 nm laser for 4000 s, the phosphorescence intensity of the sensing film remained above 95%, indicating excellent resistance to photobleaching. Moreover, the presence of ions did not affect the performance of the film. When the pH value was below 8, the Pt/TE-MTS film exhibited excellent performance in glucose detection. Additionally, the Pt/TE-MTS film demonstrated high stability and robustness in diverse environments, and the sensitivity for GC detection was not reduced. A visual GC detection system based on LabVIEW software (version: 2018) was developed to facilitate the application of the film, enabling real-time automatic GC measurement. Glucose injections with known GCs were tested using the system, and the relative errors obtained were consistently lower by 3%. In conclusion, the glucose sensing system based on the Pt/TE-MTS film exhibits high stability and accuracy for glucose quantification in diverse environments.

## Figures and Tables

**Table 1:** Real GCs, OP values, measured GC values and the calculated relative errors.

[*G*] of Samples (μM)	OP Value	Tested [*G*] (μM)	Errors (%)
100	0.91	98	2.3
250	0.79	256	2.7
400	0.68	407	1.9
550	0.58	538	2.5
700	0.47	695	2.8

## Data Availability

The data presented in this study are available on request from the corresponding author.
